# Polyfunctional Fc Dependent Activity of Antibodies to Native Trimeric Envelope in HIV Elite Controllers

**DOI:** 10.3389/fimmu.2020.583820

**Published:** 2020-09-30

**Authors:** Sanket Kant, Ningyu Zhang, Alexandre Barbé, Jean-Pierre Routy, Cécile Tremblay, Réjean Thomas, Jason Szabo, Pierre Côté, Benoit Trottier, Roger LeBlanc, Danielle Rouleau, Marianne Harris, Franck P. Dupuy, Nicole F. Bernard

**Affiliations:** ^1^Research Institute of the McGill University Health Centre Montreal, Montreal, QC, Canada; ^2^Division of Experimental Medicine, McGill University, Montreal, QC, Canada; ^3^Infectious Diseases, Immunology and Global Health Program, Research Institute of the McGill University Health Centre, Montreal, QC, Canada; ^4^Faculté de Médecine de l’Université de Lille Henri Warembourg, Lille, France; ^5^Ophthalmology Department, Lille University Hospital, Lille, France; ^6^Division of Hematology, McGill University Health Centre, Montreal, QC, Canada; ^7^Chronic Viral Illness Service, McGill University Health Centre, Montreal, QC, Canada; ^8^Centre de Recherche du Centre Hospitalier de l’Université de Montréal, Montreal, QC, Canada; ^9^Départment de Microbiologie Infectiologie et Immunologie, Université de Montréal, Montreal, QC, Canada; ^10^Clinique Médicale l’Actuel, Montreal, QC, Canada; ^11^Clinique de Médecine Urbaine du Quartier Latin, Montreal, QC, Canada; ^12^Clinique Médicale Opus, Montreal, QC, Canada; ^13^British Columbia Center for Excellence in HIV/AIDS, Vancouver, BC, Canada; ^14^Division of Clinical Immunology, McGill University Health Centre, Montreal, QC, Canada

**Keywords:** HIV, antibody dependent functions, ADCC, HIV reservoir, Elite controllers, HIV^+^ plasma, Viral controllers, HIV envelope conformation

## Abstract

Antibody dependent (AD) functions such as AD cellular cytotoxicity (ADCC) were associated with lower viral load (VL) in untreated HIV progressors and protection from HIV infection in the modestly protective RV144 HIV vaccine trial. Target cells used to measure ADCC, AD complement deposition (ADCD), and AD cellular trogocytosis (ADCT) have been either HIV envelope (Env) gp120-coated CEM.NKr.CCR5 cells or HIV infected cell cultures. In HIV infected cell cultures, uninfected bystander cells take up gp120 shed from infected cells. Both gp120-coated and gp120+ bystander cells expose CD4 induced (CD4i) epitopes, which are normally hidden in native trimeric Env expressed by genuinely HIV infected cells since Nef and Vpu downmodulate cell surface CD4. Antibody dependent assays using either of these target cells probe for CD4i Abs that are abundant in HIV^+^ plasma but that do not recognize HIV-infected cells. Here, we examined ADCC, ADCD, and ADCT functions using a target cell line, sorted HIV-infected cell line cells, whose HIV infection frequency nears 100% and that expresses HIV Env in a native trimeric closed conformation. Using sorted HIV-infected cells (siCEM) as targets, we probed the binding and AD functions of anti-gp120/Env Abs in plasma from HIV-infected untreated progressor (UTP, *n* = 18) and treated (TP, *n* = 24) subjects, compared to that in Elite controllers (EC, *n* = 37) and Viral Controllers (VC, *n* = 16), which are rare subsets of HIV-infected individuals who maintain undetectable or low VL, respectively, without treatment. Gp120-coated beads were used to measure AD cellular phagocytosis. Equivalent concentrations of input IgG in plasma from UTPs, ECs, and VCs supported higher levels of all AD functions tested than plasma from TPs. When AD activities were normalized to the concentration of anti-gp120/Env-specific Abs, between-group differences largely disappeared. This finding suggests that the anti-gp120/Env Abs concentrations and not their potency determined AD functional levels in these assays. Elite controllers did differ from the other groups by having AD functions that were highly polyfunctional and highly correlated with each other. PCR measurement of HIV reservoir size showed that ADCC activity was higher in ECs and VCs with a reservoir size below the limit of detection compared to those having a measurable HIV reservoir size.

## Introduction

The HIV vaccines tested thus far have been designed to induce cellular and/or humoral immune responses to HIV (1–4). Although a central goal of HIV vaccines is to generate neutralizing antibodies (nAbs), their induction *in vivo* has proven to be challenging (5–7). Out of the seven HIV vaccine trials conducted to date, only the RV144 trial showed significant, though moderate, success in protecting against HIV infection (4). Protection was not associated with the induction of vaccine-specific broadly neutralizing antibodies (BnAbs) or cytotoxic CD8^+^ T-cells (8). Rather, the binding of immunoglobulin G (IgG) antibodies (Abs) to the V1/V2 loop of HIV Envelope (Env) correlated with protection while the binding of IgA Abs to Env inversely correlated with protection (8, 9). In secondary analyses, high levels of antibody dependent (AD) cellular cytotoxicity (ADCC) also correlated with HIV protection in patients with low levels of plasma anti-Env IgA Abs (8, 10, 11). These findings raised interest in investigating the role of ADCC and other AD function activities in HIV control.

HIV Env is the only viral gene product expressed on the surface of infected cells and therefore represents the main target for HIV-specific Abs able to trigger Fc-dependent functions (12). Most investigations of HIV Env directed AD functions have used target cells coated with monomeric recombinant HIV Env gp120 (rgp120) (13–19). The monomeric rgp120 on these cells exposes gp120 epitopes that are normally hidden inside the native trimeric Env expressed on genuinely HIV infected cells. These epitopes are called CD4-induced (CD4i) epitopes as they are unveiled by the interaction of trimeric Env with cell surface CD4 on infected CD4^+^ cells. Furthermore, the interaction of rgp120 with the target cell’s surface CD4 receptor occludes the CD4-binding site (CD4bs) epitopes on the gp120 molecule (20). The Env CD4bs epitopes are highly conserved and Abs to these epitopes are among the most potent BnAbs (21–23). HIV infected cells have also been used as target cells for AD function assays (24–27). In HIV infected cell cultures only a fraction of CD4^+^ T cells are truly infected (28, 29). The infected cells shed gp120, which binds CD4 on uninfected bystander cells (20, 27). The interaction of shed gp120 with CD4 on bystander cells not only occludes CD4bs epitopes but also opens the Env conformation exposing CD4i epitopes (20, 27). Anti-Env Abs present in HIV^+^ plasma bind these bystander cells preferentially leading to the targeting of healthy bystander cells rather than HIV infected cells by AD functions (30). The potential pathogenicity of Abs to CD4i epitopes is illustrated by the finding that they are positively associated with mother-to-child HIV transmission and negatively associated with HIV-infected infant survival (31).

In HIV infected cells, CD4 is downregulated from the cell surface by HIV Nef and Vpu (32, 33). Unliganded HIV Env remains in a closed conformation with hidden CD4i epitopes (34). Upon interaction with cell-surface CD4 or CD4 mimetics, the conserved regions buried in the native Env trimer are exposed and targeted by ADCC- and AD complement deposition (ADCD)-mediating CD4i Abs (34–39). Target cells used for AD function assays should present HIV Env in the conformation it assumes in *in vivo* infected cells in which Nef and Vpu downregulate CD4 such that Env remains unliganded and in its native trimeric conformation (35).

Antibody dependent cellular cytotoxicity-competent Abs from vaccinees enrolled in the RV144 trial were blocked by Env C1 region-specific A32 monoclonal Ab (mAb) Fab fragments. Thus, the epitopes targeted by the ADCC competent Abs induced by the RV144 vaccine regimen were to CD4i epitopes (10, 40). In addition to Abs with ADCC activity, the vaccine used in the RV144 trial induced anti-HIV gp120-specific Abs able to activate the complement cascade and bind to Fc-receptors on monocytes to induce AD cellular phagocytosis (ADCP) and AD cellular trogocytosis (ADCT) (41, 42).

Elite controllers (ECs) are a rare subset (0.3–1%) of HIV infected individuals (43), who spontaneously control HIV viral load (VL) without treatment (44, 45). Elite controllers who maintain VLs below the limit of detection of standard VL assays and high CD4^+^ T-cell counts represent examples of a functional cure. Studying immune factors responsible for such control in ECs may uncover immune correlates responsible for HIV control that will guide strategies aimed at replicating this EC phenotype in HIV-infected progressors. Some HIV-infected individuals, known as Viral controllers (VC), maintain VLs at low but detectable levels without treatment (44).

We previously described the generation of a sorted HIV-infected cell line (siCEM) expressing HIV Env in a native, trimeric, closed conformation (27). This cell line was used to quantify the concentration of anti-Env specific Abs in plasma from HIV-infected ECs, untreated progressors (UTPs) and treated subjects (TPs) (46). We showed that HIV^+^ plasma contained Abs able to recognize Env on siCEM cells, though at lower concentrations than that of Abs binding to the open conformation of Env exposing CD4i epitopes (27). Here, we used siCEM target cells to determine the relative concentration of Abs specific for the closed conformation of Env that were ADCC-, ADCD-, and ADCT-competent. We report that plasma from UTPs, TPs, ECs, and VCs contained Abs recognizing Env on siCEM cells that mediated these three AD functions. We also showed that EC and VC subjects with superior ADCC function achieved an undetectable HIV viral reservoir size while those with inferior AD functions maintained quantifiable HIV reservoirs.

## Materials and Methods

### Ethics Statement

This research study was approved by the Institutional Review Boards of the Comité d’Éthique de la Recherche du Centre Hospitalier de l’Université de Montréal (Project Identification Code 17-096) and the Research Ethics Committee of the McGill University Health Centre (Project Identification Code 2018-4505). It was conducted according to the principles expressed in the Declaration of Helsinki. Written informed consent for the collection of each individuals’ specimens and subsequent analyses using these samples was obtained from all study subjects.

### Study Subjects

In this study, we used plasma as a source of Abs from 4 groups of HIV^+^ individuals in the chronic phase of infection. Untreated progressor (*n* = 18) were treatment-naïve individuals with VLs > 10,000 copies of HIV RNA per ml (c/ml) of plasma and CD4^+^ T-cell counts <400 cells/ml. Treated subject (*n* = 24) were on combined anti-retroviral therapy (cART) for at least one year with VLs < 50 c/ml of plasma and CD4^+^ T-cell counts >400 cells/ml. Untreated progressors and TPs were enrolled in the Montreal Primary HIV Infection (PHI) Cohort (47, 48). Elite controllers (*n* = 37) were treatment-naïve persons having VLs < 50 c/ml plasma and CD4^+^ T-cell counts >400 cells/ml. VCs (*n* = 16) were treatment-naïve individuals with VLs < 3000 c/ml of plasma and CD4^+^ T-cell counts >400 cells/ml. Elite controllers and VCs were enrolled in the Canadian Cohort of HIV Infected Slow Progressors (48).

### Total IgG ELISA

Total plasma IgG was quantified using a human IgG ELISA quantification kit (Bethyl Laboratories, Montgomery, TX) as per manufacturer’s instructions. Total IgG concentrations were tested in duplicate. The mean value of duplicate IgG concentrations in mg/ml was used to determine the volume of each plasma sample to test so that equivalent quantities of IgG were used to measure the concentration of anti-gp120 and anti-Env Abs and the AD functions of these Abs.

### SiCEM Target Cells

sorted HIV-infected cells were generated as previously described (27, 46). Briefly, CEM.NKr.CCR5 (CEM), a CD4^+^ CCR5^+^ T-cell line was infected with NL4-3-BaL-IRES-HSA, a fully replication-competent HIV-1 virus encoding Env from HIV BaL accession # AY426110 and expressing viral *Nef* under the influence of an internal ribosome entry site (IRES). The virus also encoded murine heat stable antigen (HSA; mCD24), which was cell-surface expressed and used to sort for infected cells (49). Heat stable antigen^+^ cells were sorted using a BD FACS Aria instrument (BD Biosciences, Mississauga, ON, Canada) (27, 46). Sorted cells were expanded in culture and stained for virus-mediated downregulation of CD4, expression of HSA and expression of closed conformation HIV Env (27, 46).

### Gp120- and Env-Specific IgG Quantification

The quantification of plasma Abs binding to a rgp120-coated ELISA plate [plate-based ELISA (PBE)] and to siCEM cells has been described previously (46, 50). The rgp120 used in the PBE and to coat CEM cells was derived from HIV-1 BaL gp120 (Accession # AAA44191.1 and was obtained through the NIH AIDS Reagent Program, Division of AIDS, NIAID, NIH. All plasma samples were heat inactivated before use. Multiple dilutions of subject plasma were tested in duplicate. Values that fell within the linear range of a standard curve generated by binding a positive control sample of anti-HIV immunoglobulin (HIVIG; a pool of polyclonal IgG isolated from HIV-infected donors obtained through the NIH AIDS Reagent Program, Division of AIDS, NIAID, NIH: from NABI and NHLBI) were used to calculate the relative concentration of anti-gp120 specific Abs in μg/ml relative to HIVIG. We quantified the gp120- and Env- specific Abs for 16 VCs in addition to the values previously reported for UTPs, TPs and ECs (46). IgG concentrations for each group were reported as medians [interquartile ranges (IQR)]. Negative controls included no plasma, a pool of plasma from HIV uninfected donors prepared in-house and IgG from HIV uninfected human serum (Sigma-Aldrich, St Louis, MO, United States). Binding results generated by these three negative control conditions were indistinguishable from each other (Supplementary Figure 1).

### Antibody-Dependent Cellular Phagocytosis

Antibody-dependent cellular phagocytosis activity of HIV^+^ plasma Abs from the four subject groups was assessed as described elsewhere (13, 51). Briefly, rgp120 was biotinylated using the EZ-Link^®^ Micro Sulfo-NHS-LC-Biotinylation Kit (Thermo Fisher Scientific, Burlington, ON, Canada) as per manufacturer’s instructions. One microgram of biotinylated-rgp120 was incubated with 1 μl of 1 μm fluorescent neutravidin beads (Thermo Fisher Scientific) for 2 h at 37°C. The beads were washed twice with 1 ml of phosphate buffered saline (PBS, Wisent Inc., St-Jean-Baptiste, QC, Canada); 0.1% bovine serum albumin (BSA, Sigma-Aldrich) to remove any unbound rgp120. Rgp120-conjugated beads were resuspended to a final dilution of 10 μl of beads in 1 ml PBS; 0.1% BSA. For the ADCP assay, 10 μl of total plasma IgG at concentrations of 2 and 100 μg/ml IgG in PBS was added in triplicate to separate wells of a 96-well V-bottomed microtiter plate (Sarstedt Inc., Montreal, QC, Canada) with 10 μl of diluted rgp120-coated beads for 2 h at 37°C, in a humidified, 5% CO_2_ incubator. After washing twice with PBS, 200 μl of THP-1 cells at 1.25 × 10^5^/ml RPMI-1640 media; 10% fetal bovine serum (FBS); 2 mM l-glutamine; 100 IU/ml penicillin; 100 μg/ml streptomycin (R10) (all from Wisent Inc.) was added to each well for 3 h at 37°C in a humidified, 5% CO_2_ incubator. Wells were then fixed with PBS; 2% FBS; 2% paraformaldehyde (PFA; Santa Cruz Biotechnology, Dallas, TX, United States). The positive control in this assay was HIVIG and it was used at the same concentrations as that of the total IgG in subject plasma quantified in section “Total IgG ELISA.” Cells were acquired using a high-throughput system (HTS) with a BD LSR Fortessa X20 (BD Biosciences, Mississauga, ON, Canada) instrument. All the results were analyzed using FloJo v10 software (Tree Star, Inc., Ashland, OR). The background phagocytic activity of the THP-1 cells was measured in wells containing PBS alone. Result were calculated as a phagocytic score (PS), where PS = (% of fluorescent THP-1 cells) × (mean fluorescent intensity; MFI of THP-1 cells). The no plasma background PS was subtracted from each subject’s PS. The partial area under the curve (pAUC) was calculated for PS using two concentrations of plasma and HIVIG IgG using the formula [(Y1 + Y2)/2] × (X1 - X2) where X1 and X2 were the concentrations of total IgG that generated the PSs (Y1 and Y2) that were on the linear range of the standard curve of the functional read out generated by these input IgG concentrations. Each sample’s pAUC PS was then divided by the pAUC PS of HIVIG to account for inter-plate and/or inter-assay variability. ADCP values for each group were reported as median (IQR).

### Antibody-Dependent Complement Deposition

The ADCD assay was performed as described elsewhere (13, 52, 53) with the following modifications. The target cells used were siCEM cells. 50 μl of siCEM cells at 10^6^ cells/ml RPMI were plated in duplicate into the wells of a 96-well V-bottomed microtiter plate with 50 μl of 100 and 500 μg/ml of plasma IgG for 20 min at room temperature (RT). Plasma in acid citrate dextrose anticoagulant from an HIV-negative healthy control donor, diluted 1:10 in veronal buffer (Boston BioProducts, Ashland, MA); 0.1% gelatin (Thermo Fisher Scientific) was used as a source of complement. A volume of 50 μl of diluted complement was added to each well for 20 min at 37°C, in a humidified 5% CO_2_ incubator. The reaction was stopped by washing the wells twice with 150 μl of 15 mM EDTA (Thermo Fisher Scientific). Complement deposition was detected by adding 50 μl of FITC-conjugated, mouse anti-human anti-C3b Ab (Cedarlane, Burlington, ON, Canada) diluted 1:50, for 20 min at 4°C. Cells were fixed, acquired on an LSR Fortessa X20 instrument and results were analyzed as described for the ADCP assay. The same HIVIG concentrations as used for subject plasma were included in each plate as positive controls. Background complement activity was measured in wells with no Abs. The ADCD score was calculated as the (% of C3b^+^ siCEM cells) × (MFI of C3b^+^ siCEM cells). The background score was subtracted from the scores generated by test plasma. As for ADCP, the pAUC was calculated for the ADCD score for the two concentrations of plasma and HIVIG IgG. Individual pAUCs for test samples were normalized to the pAUC of HIVIG by dividing the test results by the results for HIVIG present on the same 96-well plate to account for any inter-plate and/or inter-assay variability. ADCD values for each group were reported as median (IQR).

### Antibody-Dependent Cellular Cytotoxicity

The ADCC assay measures the cytolysis of siCEM target cells (T) by natural killer (NK) cells in the presence of Abs in HIV^+^ plasma (27). Sorted HIV-infected cells were labeled with cytosol-staining carboxyfluorescein succinimidyl ester (CFSE; Thermo Fisher Scientific) dye as per manufacturer’s instructions (27, 46). Subject plasma and HIVIG was diluted to 50 μg/ml and 500 μg/ml IgG in R10. Fifty microliter of the diluted plasma IgG was incubated in duplicate with 50 μl of CFSE^+^ siCEM cells at 2 × 10^6^cells/ml of R10 in the wells of a 96-well V-bottomed microtiter plate for 20 min at RT in the dark. Peripheral blood mononuclear cells (PBMCs) cells from an HIV seronegative, healthy leukapheresis donor were thawed and rested overnight at 37°C in a humidified, 5% CO_2_ incubator. NK effector cells (E) were enriched from these PBMCs by negative selection using magnetic beads (EasySep^TM^ Human NK Cell Enrichment Kit; STEMCELL Technologies, Vancouver, BC, Canada) as per the manufacturer’s instructions. After selection, the average purity of NK cells was 93.3%. Hundred microliter of NK E cells at 5 × 10^5^ cells/ml of R10 were added to each well to obtain a final E:T of 5:1. Wells were centrifuged for 1 min at 300 × *g* and incubated for 1 h at 37°C in a humidified, 5% CO_2_ incubator. After washing with 150 μl of 1x Annexin V (AnV) binding buffer (BD Biosciences), cells were stained with 100 μl of AnV stain (BD Biosciences) diluted 1:100 in in 1x AnV buffer for 10 min at RT in the dark. Plates were washed once in AnV buffer and resuspended in 100 μl of AnV buffer for acquisition. Results were analyzed as described for the ADCP assay. In each plate, equivalent concentrations of HIVIG IgG as in the plasma test samples and a no Ab negative control were included. Antibody dependent cellular cytotoxicity activity was defined as the average of the frequency (%) of T that were AnV^+^ after background subtraction. A pAUC was calculated for the 2 concentrations of plasma and HIVIG IgG. Subject pAUCs were normalized to the pAUC of HIVIG to account for any inter-plate and/or inter-assay variability. Antibody dependent cellular cytotoxicity values for each group were reported as median (IQR).

### Antibody-Dependent Cellular Trogocytosis

The ADCT assay used in this study was an adaptation of the rapid fluorescence ADCC (RFADCC) assay, which measures the transfer of the cell-surface membrane dye, PKH-26, from target cells to monocyte effector cells (14, 54). sorted HIV-infected cells were labeled with PKH-26 as previously described (27, 46). Subject plasma and HIVIG were diluted to 50 and 500 μg/ml IgG in R10. Fifty microliter of diluted plasma were added in duplicate to the wells of a 96-well V-bottomed microtiter plate containing 50 μl of PKH-26^+^ siCEM cells at 2 × 10^5^ cells per ml of R10 (T) for 20 min at RT in the dark. Thawed and rested PBMCs from an HIV-negative healthy control donor was used as effector cells (E). Hundred microliter of E at 3 × 10^6^ cells/ml in R10 were added to each well to obtain a final E:T of 30:1. The wells were centrifuged at 300 × *g* for 1 min and incubated for 1 h at 37°C in a humidified, 5% CO_2_ incubator. After the coculture, cells were washed with PBS; 2% FBS. To measure the ADCT activity of monocytes, each well was stained with 50 μl of Live/Dead stain (Thermo Fisher Scientific) diluted 1:500 and Brilliant Violet (BV) 785-conjugated anti-human CD14 Ab (BioLegend, San Diego, CA) diluted 1:50 in PBS; 2% FBS buffer for 20 min in a 4°C. Wells were washed once with PBS; 2% FBS, fixed, acquired and results were analyzed as described for the ADCP assay. Each plate included HIVIG positive control samples at the same IgG concentrations as test plasma and negative control wells with no plasma Abs. Antibody-dependent cellular trogocytosis activity was measured as the mean% of live CD14^+^ PKH-26^+^ monocytes. The mean% of PKH-26^+^ monocytes in the no Ab control was used for background subtraction. We calculated the pAUC for the% of live, CD14^+^ PKH-26^+^ monocytes for the 2 IgG concentrations for each subject plasma and HIVIG. Values for test plasma were normalized by dividing these results by the pAUC ADCT of HIVIG to account for inter-plate and/or inter-assay variability. Antibody-dependent cellular trogocytosis values for each group were reported as median (IQR). The ImageStream^®^ images in Supplementary Figure 2 show that the% of PKH-26^+^ monocytes were not due to doublets of PKH-26^+^ T and CD14^+^ monocytes but rather to the transfer of PKH-26^+^ membrane components from siCEM cells to CD14^+^ monocytes.

### Quantification of Latent HIV Reservoirs

The HIV reservoir size was measured using the integrated HIV DNA PCR assay described elsewhere (55, 56). Cryopreserved subject PBMCs were thawed in R10 before isolating CD4 cells by negative selection (EasySep^TM^ human CD4^+^ T cell negative enrichment kit, STEMCELL Technologies) as per manufacturer’s instructions. Post-isolation, 10^5^ enriched cells were stained with Live/Dead stain (Thermo Fisher Scientific), BV-785-conjugated anti-human CD3 (clone: OKT3; BioLegend), VioBlue-conjugated anti-human CD4 (clone: REA623) and PE-Vio770 anti-human CD8 antibody (clone: REA734) (both from Miltenyi Biotec, Auburn, CA) to ascertain the purity of CD4^+^ T cells, which averaged 96.8%. Enriched CD4 cells were lysed and stored frozen until use. The integrated DNA PCR assays were performed in triplicate and reservoir quantification was calculated as follows:

(copies of integrated HIV DNA/10^6^ CD4 cells) = [(copies of HIV as determined by PCR)/(CD3 copies as determined by PCR)] × 1,000,000 cells.

## Statistics

Microsoft Excel, GraphPad Prism v7 (GraphPad Software, Inc., San Diego, CA) and RStudio v1.2.5001 (RStudio: Integrated Development for R. RStudio, Inc., Boston, MA) were used for statistical analyses and graphical presentation. The statistical significance of between-group differences was determined using non-parametric, Kruskal-Wallis tests with Dunn’s post tests. *p*-values < 0.05 were considered significant. The statistical significance of correlations and the respective plots between AD function assays and AD function assays with Ab concentrations were assessed using non-parametric Spearman’s correlation tests in Rstudio v1.2.5001.

## Results

### Plasma From UTPs and HIV Controllers Have Higher HIV-gp120/Env-Specific Abs Concentration Than TPs

**FIGURE 1:**
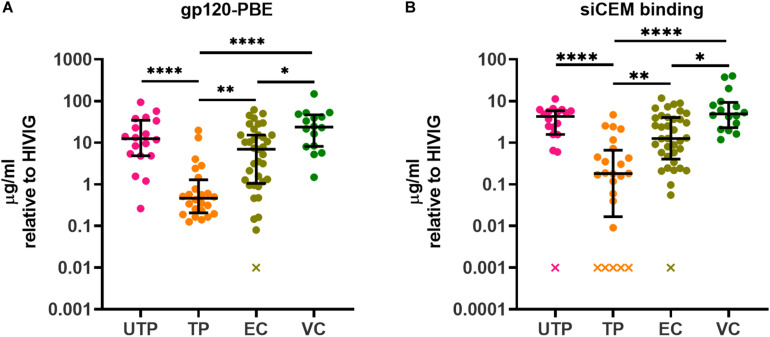
Quantification of HIV gp120- and Envelope (Env)-specific antibodies (Abs) in plasma from HIV^+^ subjects. The concentration of gp120/Env-specific Abs in plasma from four groups of HIV^+^ subjects relative to HIVIG was assessed using panel **(A)** an ELISA assay in which plates were coated with recombinant gp120 (gp120) and **(B)** by measuring binding to native, closed conformation Env on sorted HIV-infected CEM.NKr.CCR5 (siCEM) cells by flow cytometry. “x” denotes sample(s) for which Abs concentrations were below the limit of detection. Each point represents results generated by the plasma of a single individual. Lines and error bars though each data set represent medians and inter-quartile ranges (IQRs). Data from 18 UTPs, 24 TPs, 37 ECs and 16 VC are used to prepare panels A and B. Kruskal-Wallis tests with Dunn’s multiple comparisons post test were used to assess the significance of between-group differences. ^∗^*p* < 0.05, ^∗∗^*p* < 0.01, ^****^p < 0.0001. HIVIG, a pool of plasma from HIV infected subjects; gp120-PBE, gp120 plate-based ELISA; UTP, untreated progressors; TP, HIV infected subjects in chronic phase infection on antiretroviral therapy; EC, Elite Controllers; VC, Viral Controllers.

Of the four subject groups tested, UTPs had the highest concentration of total plasma IgG with a median (IQR) of 15.41 (12.45, 19.38) mg/ml, which was significantly higher than the 9.93 (8.01, 12.22), 10.24 (8.22, 15.27), and 8.95 (6.46, 13.78) mg/ml of total plasma IgG observed in TPs, ECs and VCs, respectively (Supplementary Figure 3). These results likely reflect hypergammaglobulinemia in this chronically infected, untreated population (57, 58). The AD function assays were performed using equivalent concentrations of plasma IgG. In addition, in this study to determine the relationship between the concentrations of specific anti-gp120- and anti-Env Abs and their individual AD functions, we quantified Abs specific to these antigens using a PBE with wells coated with monomeric gp120 and by flow cytometry using siCEM cells expressing the native trimeric Env, respectively. We previously showed that UTPs and ECs had similar levels of anti-gp120- and anti-Env-specific Abs (46). Figures 1A,B display the concentrations of Abs binding to rgp120 using the PBE and closed conformation Env on siCEM cells relative to a standard curve of serial dilutions of known concentrations of pooled IgG isolated from HIV-infected donors (HIVIG). Since the exact amount of Abs binding to rgp120 and trimeric Env in the HIVIG is unknown, our results for subject plasma Abs are reported as “μg/ml normalized to HIVIG”. We were unable to quantify anti-gp120 Abs levels for 1 EC in the PBE assay and for 1 UTP, 5 TP and 1 EC in the siCEM binding assay because these values were below the limit of detection. In addition, we quantified plasma from 16 VCs in this study. VCs had higher levels of both anti-gp120 and anti-Env Abs than did TPs and ECs that did not differ significantly from those measured in UTPs. Overall, TPs had lower levels of anti-gp120- and anti-Env-specific Abs than did UTPs, ECs and VCs.

## HIV-Specific Functions in UTPs, TPs, ECs, and VCs

**FIGURE 2:**
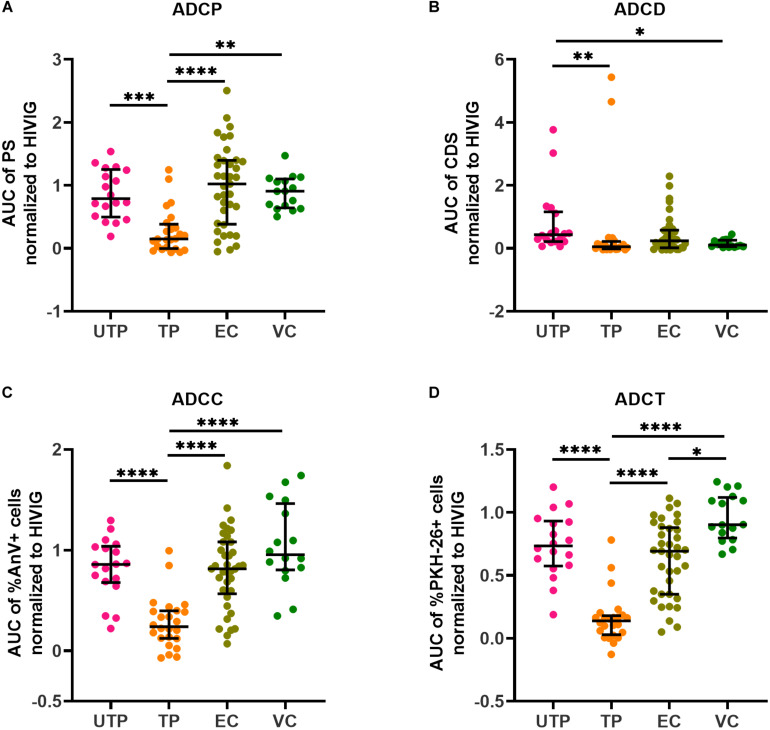
Quantification of Ab-dependent (AD) functions in HIV^+^ plasma. HIV^+^ plasma from UTPs, TPs, ECs and VCs were tested for **(A)** AD cellular phagocytosis (ADCP), **(B)** AD complement deposition (ADCD), **(C)** AD cellular cytotoxicity (ADCC), and **(D)** AD cellular trogocytosis (ADCT) as described in the methods. Each point represents results generated by the plasma of a single individual. Lines and error bars though each data set represent medians and IQRs. Data from 18 UTPs, 24 TPs, 37 ECs and 16 VC are used to prepare these four panels. Kruskal–Wallis tests with Dunn’s multiple comparisons post test were used to assess the significance of between-group differences. ^∗^*p* < 0.05, ^∗∗^*p* < 0.01, ^∗∗∗^*p* < 0.001, ^****^p < 0.0001. All results shown were background subtracted and were normalized to the concentrations of internal HIVIG positive controls tested at the same time as test samples. AUC, partial area under the curve; PS, phagocytic score; CDS, complement deposition score; %AnV + cells, frequency of AnnexinV^+^ siCEM cells; %PKH-26 + cells, frequency of PKH-26^+^ cells.

We performed four AD function assays that measured Fc-dependent functions of plasma HIV gp120/Env-specific Abs. The read outs for the four assays measuring AD function were (1) the pAUCs of the PS for ADCP using biotinylated rgp120 coupled to neutravidin beads, (2) the pAUCs of the complement deposition score (CDS) for ADCD, (3) the pAUCs of the% of AnV^+^ siCEM cells for ADCC, and (4) the pAUCs of the% of PKH-26^+^ monocytes for ADCT. The target cells for ADCD, ADCC, and ADCT were siCEM cells. Supplementary Figure 4 shows the non-normalized pAUC results for these four assays performed on plasma from the four subject groups as well as the plate to plate variation in the pAUC generated by HIVIG. Figure 2 shows pAUC results for these four AD functions after normalization to the pAUC of the HIVIG standard curve present in the same 96-well plate in which each test was performed. In all four AD function assays, plasma from UTPs, ECs and VCs did not differ significantly from each other in their response score with the exceptions of UTPs having higher ADCD activity than VCs and VCs having higher ADCT activity than ECs (Figures 2A–D). The ADCP functional score of anti-gp120-specific Abs and the ADCC and ADCT functional score of anti-Env-specific Abs in plasma from the UTP, EC, and VC groups were higher than those reported for TPs. So too the ADCD functional score of the anti-Env-specific Abs in plasma from UTPs was higher than that in TPs and VCs (Figures 2A–D).

**FIGURE 3:**
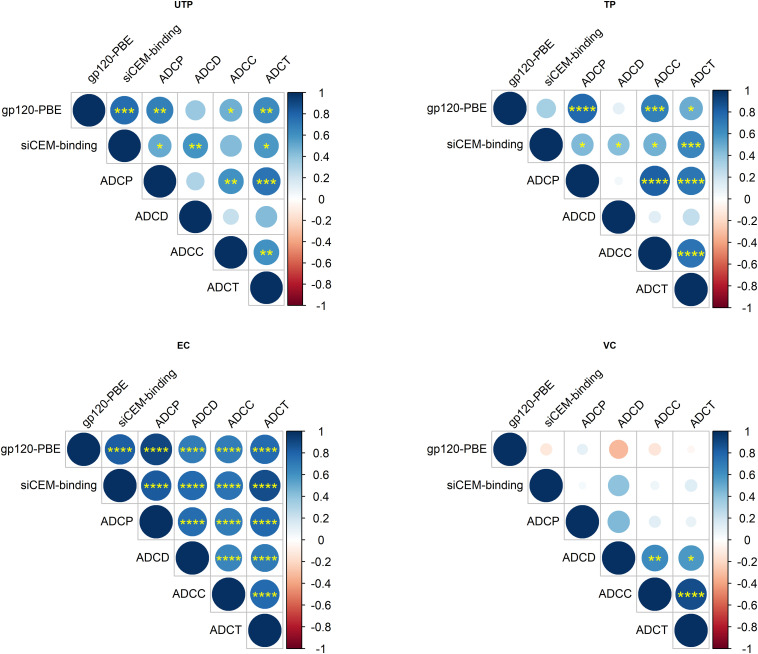
Correlation of AD functions with each other and with anti-HIV-gp120/Env Ab concentrations within HIV-infected subject groups. Correlation matrices for each pairwise combination of AD function and anti-gp120/Env-specific Abs concentrations tested in the four subject groups. Data from 18 UTPs, 24 TPs, 36 ECs and 16 VC were used to generate panel **(A)**. Data from 17 UTPs, 19 TPs, 36 ECs and 16 VC were used to generate panels **(B,C)**. The increasing strength of positive correlations are illustrated using increasing blue color depth, as depicted in the legend and by the size of each circle. The statistical significance of pairwise correlations is indicated by the number of “^∗^” symbols in each circle (*p < 0.05, **p < 0.01, ***p < 0.001, ****p < 0.0001). Empty circles represent p-values for correlative relationships that fell below the level of significance (*p* > 0.05). Shown are unadjusted *p*-values to aid in relative comparisons. Correlation coefficients were calculated using Spearman’s correlation tests and they were plotted using Rstudio v1.2.

Even though gp120-specific and Env-specific Abs from ECs had significantly higher AD functionality levels compared to TPs for but ADCD, that did not differ significantly from those in UTPs and VCs, correlation analyses revealed that these functions were more highly correlated with each other in ECs than in the other study groups (Figure 3). Overall, the functional read outs of ADCC and ADCT activity of anti-Env-specific Abs correlated with each other in all 4 study groups with correlation coefficients (*r*-values) of 0.61, 0.74, 0.77, and 0.89 for UTPs, TPs, ECs, and VCs, respectively (*p* < 0.01 for all). In ECs, all AD functions were correlated with each other with *r*-values > 0.66 (*p* < 0.0001 for all pairs). In contrast, ADCD assay scores correlated with ADCP, ADCC and ADCT results in UTPs with r-values of 0.31, 0.23, and 0.43, respectively (p < 0.05 for all comparisons). For VCs, ADCD scores correlated with ADCP, ADCC, and ADCT with *r*-values of 0.45 (*p* = 0.08), 0.64 (*p* < 0.01), and 0.58 (*p* < 0.02), respectively. In VCs, ADCP activity did not correlate significantly with any other AD functional result. Together, these results suggest that while there may not be between-group differences in the AD function of anti-Env Abs relative to HIVIG between UTPs, ECs, and VCs (Figures 2A–D), these functions were more highly correlated in ECs than in UTPs and VCs (Figure 3).

### AD Functional Scores Are Dependent on the Concentration of Abs Specific to gp120 or Env Present in HIV^+^ Plasma

While previous studies have investigated the underlying relationships between the AD functions with IgG subtypes (13) and with IgG post-translational modifications (59), input IgG concentration has rarely been controlled for, nor has between-subject variation in the concentration of Abs specific for gp120 or Env been accounted for. To address this, we first investigated whether there was a relationship between anti-gp120/Env concentrations and AD functions. Since we used gp120-coated beads in the ADCP assay, results from the ADCP assay were correlated with the concentration of Abs quantified from the gp120-coated PBE. As the target cells for ADCD, ADCC and ADCT assays were siCEM cells, the results of these assays were correlated with the concentration of anti-Env-specific Abs quantified using siCEM cells (Figure 3). ADCP levels correlated significantly with the concentration of anti-gp120-specific Abs in UTPs, TPs, and ECs (*r* = 0.68, 0.78, and 0.91, respectively; *p* < 0.01 for all) but not in VCs (*r* = 0.1; *p* > 0.05). Anti-Env Ab concentrations in HIV^+^ plasma correlated with ADCD, ADCC and ADCT activity in UTPs (r = 0.59, p < 0.01; r = 0.43, p = 0.07; r = 0.57, p < 0.05, respectively), in TP (*r* > 0.42; *p* < 0.05 for all) and in ECs (*r* = 0.77, 0.73, and 0.88, respectively; *p* < 0.0001, for all). Anti-Env Ab concentrations did not correlate significantly with any of these three AD functions in VCs (Figure 3).

**FIGURE 4:**
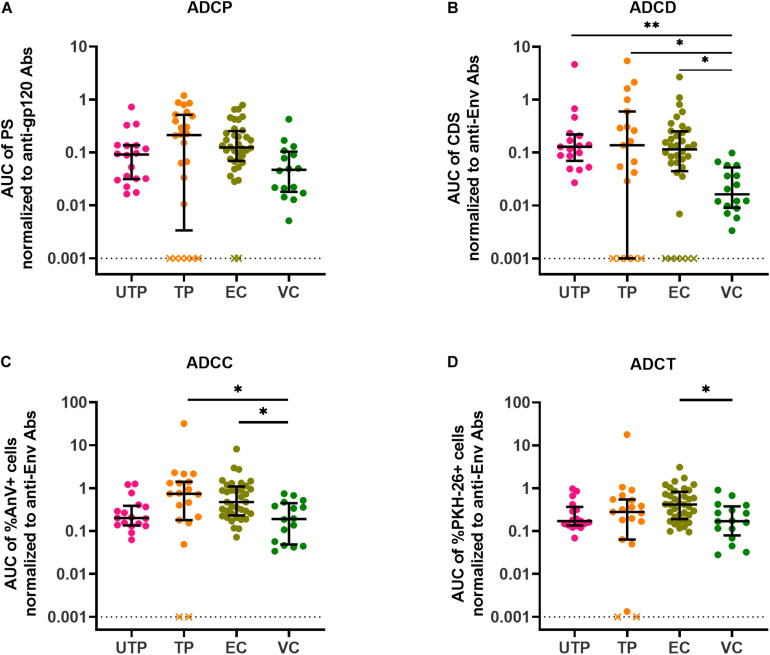
AD function results normalized to input anti-gp120/Env-specific Ab concentrations present in HIV^+^ plasma samples. **(A)** ADCP functional results were normalized to anti-gp120-specific Ab concentrations in HIV^+^ plasma quantified using the PBE assay **(B)** ADCD, **(C)** ADCC, and **(D)** ADCT functional results were normalized to anti-Env Ab concentrations in HIV^+^ plasma quantified by binding to siCEM cells. Each point represents a single subject. Lines and error bars though each data set represent medians and IQRs. Data from 18 UTPs, 24 TPs, 37 ECs, and 16 VC are used to prepare these four panels. Kruskal-Wallis tests with Dunn’s multiple comparisons post test were used to assess the significance of between-group differences. ^∗^*p* < 0.05, ^∗∗^*p* < 0.01. All results shown were background subtracted and are relative to the concentrations of an internal HIVIG positive control tested at the same time as test samples. “x” symbols represent negative values after background correction. AUC, partial area under the curve; PS, phagocytic score; CDS, complement deposition score; %AnV + cells, frequency of Annexin V^+^ siCEM cells; %PKH-26 + cells, frequency of PKH-26^+^ cells.

This prompted us to question whether anti-gp120/Env concentrations affected the level of AD functionality. To address this, we normalized each subject’s AD functional results by dividing these results by the concentration of their anti-gp120- or anti-Env-specific Abs. After normalization, variation in assay results within groups was reduced with a few exceptions and many of the statistically significant between-group differences in the four AD assays observed before normalization were no longer present. Where between-group differences were obtained post-normalization, they were driven by a few outlier data points. In summary, these results showed that HIV gp120/Env-specific Ab concentrations play an important role in contributing to variation in AD functions (Figures 4A–D). The loss of between-group difference in AD functionality also suggests that AD functional Ab potency did not differ between groups.

### Higher ADCC Function Is Observed in Individuals With Undetectable Reservoir

**FIGURE 5:**
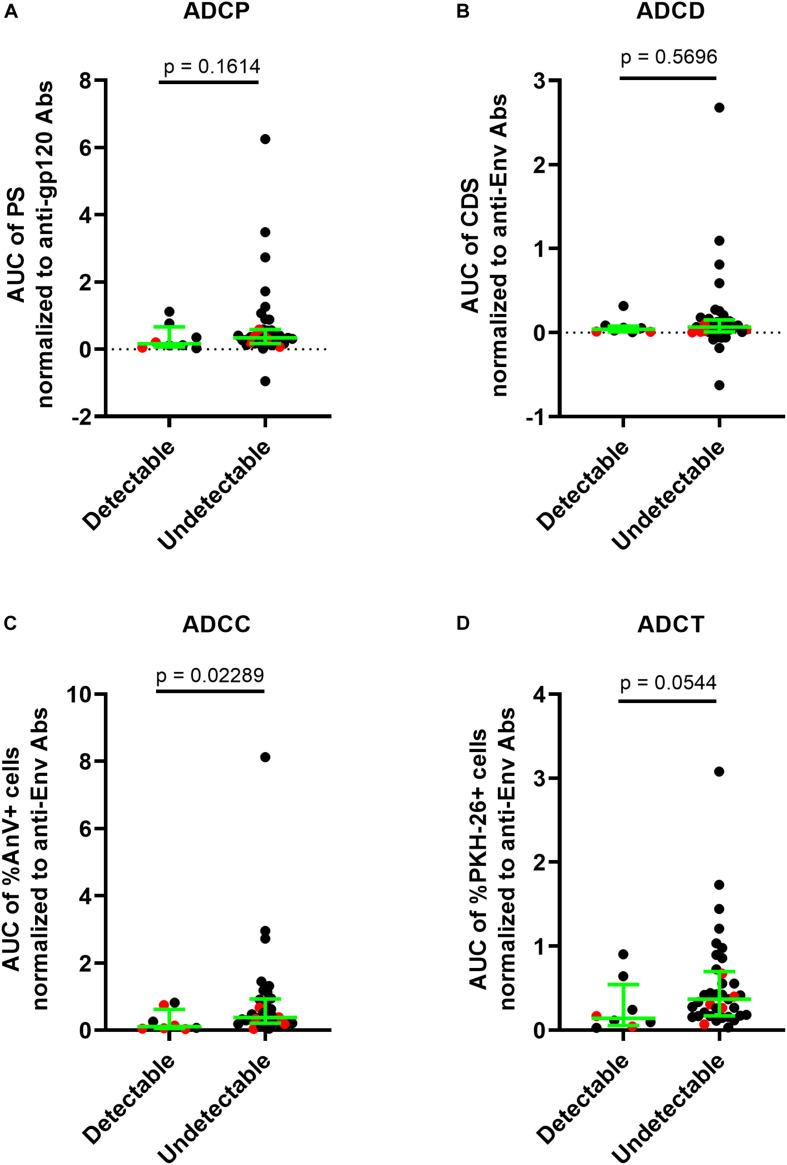
Higher ADCC function in HIV controllers is associated an HIV reservoir size below the limit of quantification. The HIV reservoir size in 30 ECs and 16 VCs was measured using the integrated HIV PCR assay. Results were stratified into a group with detectable [n = 8, (3 EC and 5 VC)] and undetectable [(n = 38, 27 (EC and 11 VC)] HIV reservoir sizes. **(A)** The *y*-axis shows the levels of ADCP function normalized to the anti-gp120-specific Ab concentrations in HIV^+^ plasma quantified using the PBE assay. **(B–D)** The y-axes show the levels of ADCD, ADCC, and ADCT functions, respectively, normalized to the anti-Env Ab concentration in HIV^+^ plasma quantified using the siCEM binding assay. Each point represents a single subject. Results for ECs are illustrated in black and those for VCs in red. Lines and error bars though each data set represent medians and IQRs. The significance of between-group differences was assessed using Mann–Whitney tests. *p*-values are depicted above the lines linking groups being compared.

ECs are untreated individuals with VLs below the limit of detection of standard VL assays, whereas VCs, who are also untreated, have low but detectable VL levels of <3000 c/ml of plasma. Given that both EC and VC groups have high levels of non-neutralizing AD functions in the range of those seen in UTP, we questioned whether AD function levels were associated with cell-associated DNA reservoir levels. We therefore quantified the HIV reservoir in ECs and VCs using the integrated HIV DNA PCR assay (55, 56). The minimum detection limit of the standard curve in the integrated HIV DNA PCR assay was 3 HIV copies per 0.1 million ACH2 cells, which carry a singly copy of integrated HIV DNA per cells. Each subject’s reservoir quantification was performed in triplicates. We distinguished the reservoir results of the subjects as “Quantifiable (≥2 of the triplicates having ≥3 HIV copies per 0.1 million CD4 cells)” and “Undetectable (<2 of the triplicates having <3 HIV copies per 0.1 million CD4 cells).” Out of the 46 subjects whose HIV reservoir could be evaluated (ECs, *n* = 30 and VCs, *n* = 16), the reservoir was quantifiable in eight subjects (ECs, *n* = 3 and VCs, *n* = 5) and the remaining samples had an HIV reservoir size below the limit of detection and were thus categorized as “undetectable.” The HIV Env-specific Ab ADCC functional score relative to HIVIG were higher in subjects with an undetectable than a detectable HIV reservoir size (*p* = 0.0229, Mann–Whitney test). We also observed a non-significant trend toward a difference in the Ab normalized ADCT, ADCP, and ADCD scores results in subjects with quantifiable versus undetectable HIV reservoir sizes of 0.1416 vs 0.3700 (*p* = 0.0544) for ADCT, 0.1607 vs 0.3391 (*p* = 0.1614) for ADCP and 0.3857 vs 0.6493 (*p* = 0.5696) for ADCD (Figures 5A–D).

## Discussion

In this study, we used siCEM cells rather than rgp120-coated CEM cells as target cells in ADCC, ADCT, and ADCD function assays. Using siCEM cells as target cells allowed us to probe for the presence of Abs in HIV^+^ plasma to a native, closed conformation of Env exposed on infected cells (27, 46). We show here that plasma from 89 of 96 (92.70%) HIV^+^ study subjects had detectable levels of Abs binding to native trimeric HIV Env on siCEM cells. Plasma from UTPs, ECs and VCs had significantly higher concentrations of Abs specific for Env on siCEM that supported these AD functions than did TPs on ART. This was also the case for ADCP activity, which was measured using rgp120-coated fluorescent beads. When ADCC, ADCT, and ACCD function results were normalized to the concentration of HIV Env-specific Abs in each subject’s plasma, between group differences in AD functions were no longer present. Between group differences in ADCP function were also lost following normalization to the concentration of anti-gp120-specific Abs in subject plasma. This indicated that variation in AD functions was directly dependent on the anti-Env or anti-gp120-specific Ab concentration and was not due to between-group differences in the potency of Abs to support these AD functions. A distinguishing feature of ECs was that their AD responses were more highly and significantly correlated to each other than those of UTPs, TPs, and VCs. We also report that individuals who had an HIV reservoir size below the limit of detection had higher AD functional responses, which achieved statistical significance for ADCC activity than those whose HIV reservoir size was quantifiable.

The results reported here for ADCC, ADCT and ADCD function of anti-HIV Env-specific Abs in HIV^+^ plasma differ in several aspects from those generated by other groups (13, 18, 19, 60). This is likely due to the target cell we used to measure these functions. Rgp120-coated cells have been used extensively as target cells in the investigation of AD functions and in immune monitoring of HIV vaccine trials (3, 10, 13, 14, 17–19, 40, 42, 59–62). Interaction of Env with CD4 exposes conserved residues that are generally hidden in Env on productively infected cells. This exposed inner domain of Env is preferentially bound by Abs to CD4i epitopes. Such epitopes are also accessible on cells infected with HIV *Nef* and/or *Vpu* deletion mutants that fail to downmodulate CD4 (35, 36). Some groups have also studied AD functions using HIV infected cell cultures (25, 26, 63). However, only a fraction of CD4 cells are infected in such cultures. Gp120 is shed from infected cells to be taken up by CD4 on uninfected bystander CD4 cells, also leading to exposure of CD4i epitopes (20). The consequence of this phenomenon is the preferential targeting by ADCC of uninfected bystander, rather than infected cells opsonized by Abs to CD4i epitopes (20, 27, 30). Thus, studying AD functions using rgp120-coated target cells, cells infected with *Nef^–^ Vpu^–^* variants of HIV or cultures in which only a fraction of the target cells are HIV infected, probes for the presence of Abs in HIV^+^ plasma that recognize CD4i epitopes of gp120/Env. To overcome this obstacle, we used siCEM cells as a target cell, a productively infected cell line that synthesizes functional Nef and Vpu able to downregulate CD4, tetherin/BST-2 and HLA-C (27, 46). In the absence of cell surface CD4, HIV Env remains in a closed conformation as confirmed by the failure of CD4i-specific Abs such as A32 and C11 to bind Env on siCEM cells (27, 46). In addition to functional viral proteins, the virus used in this model synthesises murine HSA/CD24 (mCD24) which was used to select and sort for infected cells (27, 46, 49). Thus, the use of siCEM target cells provided a unique platform for the assessment of the non-neutralizing functions of anti-Env Abs in plasma from HIV^+^ subjects. In this report, we also developed an in-house ADCC assay that measures ADCC as the frequency of apoptotic target cells as a surrogate for target cell cytolysis (27). Apoptosis is a later step in the cascade of events leading to cell death than the activity of granzyme B measured by the GranToxiLux assay (64). It differs from the RFADCC assay, which measures trogocytosis rather than ADCC activity (14, 54, 65). Lastly, our experiments controlled for the input of bulk IgG in plasma. Thus, the between-group differences in AD function assays were a result of the amount of HIV-Env/gp120-specific Abs present in each plasma sample. While the biological properties and subclass distributions of anti-gp120-specific Abs have been shown to have an influence on downstream AD functions, we observe that the amount of Env/gp120-specific Abs played a significant role in the quantification of our AD functional assay readouts.

The phase III RV144 trial was significantly, though modestly, successful in preventing HIV infection (4). This led to significant interest in investigating the role of ADCC activity and other AD functions in protection from HIV infection. Efforts to replicate RV144 vaccine-mediated protection in South Africa using a regimen analogous to the one used in the RV144 trial but with a vaccine regimen based on clade C HIV, the HVTN702 vaccine trial, failed to protect against HIV infection (66, 67). Companion phase II safety and immunogenicity vaccine trials showed that levels of anti-Env Abs induced by the clade C based vaccine regimens were as high if not higher than the levels induced by the RV144 vaccine regimen (3, 62, 68, 69). These Abs responses were tested using cells coated with monomeric rgp120 and thus, the immune monitoring strategy preferentially detected Abs to CD4i epitopes. Differences in the protection offered by this vaccine strategy in Thailand versus South Africa may be due to subtle amino acid differences between the predominant HIV strain circulating in Thailand (circulating recombinant form (CRF)01_AE) and clade C HIV in South Africa (70–72). Env from all the phylogenetic M groups possess a serine (S) at amino acid 375, except for the CRF01_AE isolates, which possesses a histidine (H) at this position (71, 72). H375 modifies the HIV Env conformation to a state closer to that of its CD4 bound conformation (71, 73). HIV^+^ plasma contains abundant Abs to CD4i epitopes (20, 27, 35–37, 74). The vaccine regimen used in the RV144 trial induced A32-blockable Abs that were specific for the gp120 inner domain. Thus, the reduced risk of infection in RV144 HIV trial recipients may be related to the induction of cluster A-specific Abs able to recognize HIV CRF01_AE-infected cells bearing an Env conformation that is uniquely already partially open. On the other hand, cluster A-specific Abs to an analogous sequence induced by a vaccine regimen that included clade C gp120 may be unable to recognize HIV infected cells in a South Africa population where clade C Env adopts a more closed conformation on infected cells. In addition, in the RV144 trial as in companion trials, Ab responses were tested using rgp120 coated cells (or HIV CRF01_AE infected target cells) exposing cluster A epitopes usually hidden inside trimeric Env of other HIV clades. This strategy may have been appropriate to detect vaccine induced Abs able to support ADCC of HIV-infected target cells in the Thai population but not in other countries where HIV CRF01_AE is not dominant. In such a setting, ADCC target cells expressing native trimeric Env in a closed conformation would have the potential to be superior to rgp120-coated target cells (or HIV CRF01_AE infected target cells) for the purpose of immune monitoring vaccine-induced Abs able to support the ADCC of genuinely HIV infected cells.

Elite controllers represent a rare population of HIV infected persons who spontaneously control HIV. There is interest in understanding the mechanisms underlying HIV control without treatment in ECs as this may guide strategies aimed at controlling VL without treatment to effect a functional cure in a broader range of HIV infected persons. Elite controllers, unlike those in any other study group, had an anti-HIV Env Ab response able to mediate AD functions that were highly correlated with each other. This has been reported by others (13). Although previous studies have not observed any significant differences between TPs, UTPs, and ECs in terms of the ability of their anti-gp120 Abs to support AD functions, these studies did report that subclass differences and glycosylation patterns could be key actors in HIV control (13, 59). Additionally, in these studies, AD non-neutralizing functions were not normalized to the amounts of HIV-gp120-specific Abs. Here, we demonstrated that the amount of the HIV gp120/Env-specific Abs play a significant role in non-neutralizing functions. Unlike UTP, whose Ab responses are likely driven by high VLs, ECs, maintained high levels of Env-specific Abs with AD functionality despite undetectable VL levels. Furthermore, although ECs and successfully treated TPs had VL levels that were <50 c/ml of plasma, they differed from each other in that ECs maintained high anti-gp120/Env levels while TPs did not. How ECs maintain high levels of anti-Env/gp120-specific Abs is not well understood. One possibility is that ECs may have a significantly higher HIV antigen-specific memory B cell response than HIV^+^ subjects on-ART (75) and untreated persons in chronic phase infection (76). Alternatively, B cells may be stimulated by a low-level of systemic HIV replication that is generally undetectable by currently available VL measuring tools (77–80). Whether ECs display a unique memory B cell phenotype that is different from other groups in our study is worth investigating (75).

A limitation of this study is the choice of HIV BaL Env in the construct used to infect CEM cells and generate siCEM cells. BaL Env is macrophage tropic, has a Tier 1 phenotype and requires little CD4 to support infection. Another limitation is the use of a single Env for these studies. Future experiments should substitute BaL Env with Envs from isolates that more closely resemble circulating viruses, transmitting/founder viruses, Envs from HIV clades other than clade B and Envs being used in vaccine constructs.

In addition to AD functions, we measured the cell-associated reservoir in 53 ECs and VCs by using the integrated HIV PCR assay (55, 56). Of these controllers, only 8 (15.07%) had a reservoir size that was detectable of which 3 individuals were ECs. When ECs and VCs were stratified into two groups depending on whether their HIV reservoir size was above or below the limit of detection of this assay, we found that AD function measures were higher in the group with an undetectable HIV reservoir size. This was the case for all AD functions though the differences only achieved statistical significance for ADCC. This finding suggests that AD function, which depends on anti-Env Ab concentration may be associated with HIV control at the level of the HIV reservoir. A limitation of this work is that the measurement of latent HIV reservoirs is challenging, particularly in HIV controllers. The quantitative viral outgrowth assay (QVOA) is considered the gold standard for reservoir quantification. However, this assay is thought to underestimate the HIV reservoir size because a single round of stimulation does not activate all CD4 cells harboring replication-competent HIV (81). The QVOA requires large numbers of isolated CD4 cells, is time consuming and technically challenging compared to PCR-based assays such as the integrated HIV DNA PCR assay used here. A drawback of the integrated HIV DNA PCR assay is that it overestimates the HIV reservoir size because it also amplifies integrated viral fragments that are not full-length sequences of replication competent HIV. Several assays have been developed to improve the quantification of cells harboring replication competent HIV. The *Tat/Rev* induced limiting dilution assay (TILDA) measures the frequency of cells with virus isolates able to transcribe multiply-spliced HIV RNA (82). Flow cytometry-based techniques such as RNA flow-FISH (83, 84) simultaneously measures *GagPol* mRNA and HIV protein double-positive cells. The HIV-Flow assay (85) measures the frequency of cells harboring HIV sequences complete enough to translate HIV p24. The flow cytometry-based assays offer advantages in characterizing the phenotype of cells harboring the reservoir but the starting number of isolated CD4^+^ cells required for these assays limits the application of these methods to subjects who have undergone leukaphereses. However, the results of the RNA flow-FISH and HIV-Flow assays are significantly correlated with the results generated using the integrated HIV DNA PCR assays (84, 85).

In summary, the study reported here used siCEM cells expressing HIV Env in its native trimeric form to probe plasma from HIV-infected subjects for the presence of Abs recognizing this Env conformation. Sorted HIV-infected cells were used to quantify Abs of this specificity in different groups of HIV infected subjects. Untreated progressors, ECs, and VCs had similar levels of Env-specific Abs that were higher than those in TPs. These Abs supported Fc-dependent functions, which were dependent on the amount of Env-specific Abs present in the plasma. A unique aspect of Abs from ECs that distinguished them from other study groups was their polyfunctional Fc-dependent activities as suggested by their highly correlated AD functions (13). A novel feature of this study is that we found that individuals with an undetectable HIV reservoir size tended to have higher AD function levels as compared to individuals with a quantifiable HIV reservoir. Future studies should be directed at characterizing the biologic properties and subclass distribution of these Abs and the B cell phenotypes of ECs, which may account for their ability to control HIV without treatment.

## Data Availability Statement

The raw data supporting the conclusions of this article will be made available by the authors, without undue reservation.

## Ethics Statement

The studies involving human participants were reviewed and approved by Institutional Review Boards of the Comité d’Éthique de la Recherche du Centre Hospitalier de l’Université de Montréal (Project Identification Code 17-096) and Research Ethics Committee of the McGill University Health Centre (Project Identification Code 2018-4505). The patients/participants provided their written informed consent to participate in this study.

## Author Contributions

SK, AB, FD, and NB contributed to the conception and design of the study. SK, NZ, AB, and FD performed the experiments and analyzed the results. SK performed the statistical analysis and prepared the figures for the manuscript. SK and NB wrote the first draft of the manuscript. J-PR, CT, RT, JS, PC, BT, RL, DR, and MH provided samples with linked clinical follow up information from the subjects participating in this study. NB supervised the project, provided administrative oversight, and obtained funding for this study. All authors read and contributed to manuscript revisions and approved the submitted version of the manuscript.

## Conflict of Interest

The authors declare that the research was conducted in the absence of any commercial or financial relationships that could be construed as a potential conflict of interest.

## Supplementary Material

Supplementary Figure 1 Frequency of Annexin V^+^ target cells generated by negative control conditions in an antibody dependent cellular cytotoxicity (ADCC) assay. The y-axis shows the frequency of Annexin V^+^ target cells generated in an ADCC assay as described in the methods section. Target cells included siCEM cells tested 3 times and HIV uninfected CEM cells (negative control). Target cells were opsonized with 50 or 500 μg/ml of IgG from the positive control HIVIG, a pool of plasma from HIV negative subjects (HIV^–^Ig #1), IgG from HIV negative serum (HIV^–^Ig #2) and no antibody. Each antibody concentration and the no antibody wells were tested in duplicate and each symbol represents the mean of duplicate determinations.

Supplementary Figure 2 Visualization of antibody dependent cellular trogocytosis (ADCT) using ImageStream^®^. Single monocytes acquire pieces of membrane from HIVIG opsonized gp120-coated CEM cells labeled with CFSE and PKH-26. **(A)** Three single gp120-coated CEM cells positive for the fluorochromes FITC (CFSE) and PE (PKH-26) but not for BV786 (CD14). **(B)** Three single monocytes as shown by the positive signal for the BV786 fluorochrome conjugated to anti-CD14, after a one-hour incubation with CFSE^+^PKH26^+^ gp120-coated CEM cells and HIVIG. Membrane (PKH-26) but not cytosolic (CFSE) material was transferred to gp120-coated CEM cells by ADCT to single monocytes as demonstrated by the absence of doublets by bright field microscopy.

Supplementary Figure 3 Quantification of total IgG in HIV^+^ plasma. Plasma samples from 18 UTPs, 24 TPs, 37 ECs and 16 VCs were quantified for total IgG using a plate-based human IgG ELISA quantification kit.

Supplementary Figure 4 Quantification of Ab-dependent (AD) functions in HIV^+^ plasma. Shown are the results generated by HIV^+^ plasma from 18 UTPs, 24 TPs, 37 ECs and 16 VCs tested for **(A)** ADCP, **(B)** ADCD, **(C)** ADCC and **(D)** ADCT as in Figure 2. Results shown were background subtracted but were not normalized to the concentrations of their internal HIVIG positive controls. Panels **(A–D)** display the results generated by HIVIG present in the 9, 5, 8 and 8 96-well plates, respectively, used to test these AD functions. AUC = partial area under the curve; PS = phagocytic score; CDS = complement deposition score; %AnV+ cells = frequency of Annexin V^+^ cells; %PKH-26+ cells = frequency of PKH-26^+^.
