# Supplementary material for: Polyfunctional Fc Dependent Activity of Antibodies to Native Trimeric Envelope in HIV Elite Controllers
Source: Front Immunol. 2020 Sep 30;11:583820. doi: 10.3389/fimmu.2020.583820 (PMC7555699; doi:10.3389/fimmu.2020.583820)

Supplementary figure 1

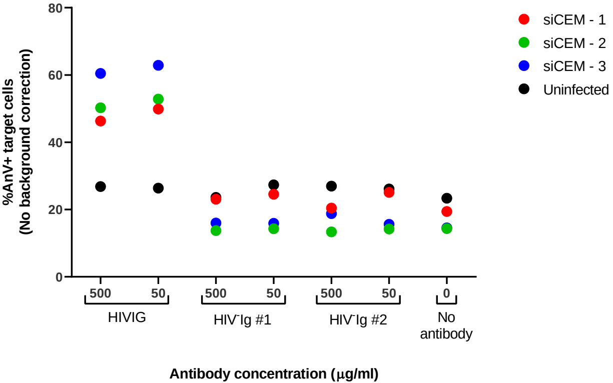

Supplementary figure 2

(A) **CD14⁻, rgp120-coated CEM cells (x3)**

(B) **CD14⁺ monocytes (x3) after co-culture with CFSE⁺ PKH-26⁺ gp120-coated CEM cells and HIVIG**

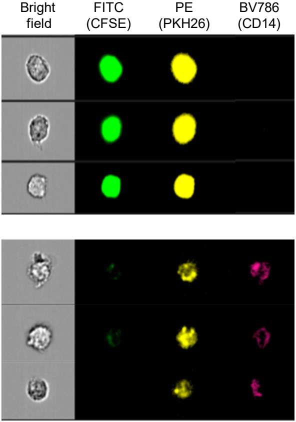

Supplementary figure 3

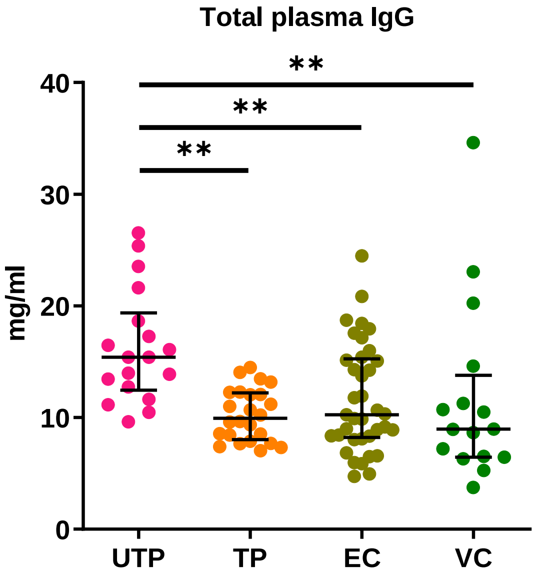

Supplementary figure 4

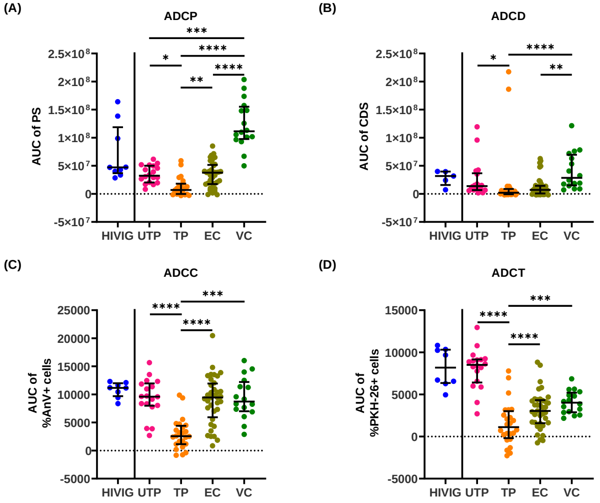

Supplement: Supplementary Figure 1 — Frequency of Annexin V+ target cells generated by negative control conditions in an antibody dependent cellular cytotoxicity (ADCC) assay. The y-axis shows the frequency of Annexin V+ target cells generated in an ADCC assay as described in the methods section. Target cells included siCEM cells tested 3 times and HIV uninfected CEM cells (negative control). Target cells were opsonized with 50 or 500 μg/ml of IgG from the positive control HIVIG, a pool of plasma from HIV negative subjects (HIV–Ig #1), IgG from HIV negative serum (HIV–Ig #2) and no antibody. Each antibody concentration and the no antibody wells were tested in duplicate and each symbol represents the mean of duplicate determinations. [file Data_Sheet_1.PDF]
